# Ovarian carcinoma in children with constitutional mutation of *SMARCA4*: single-family report and literature review

**DOI:** 10.1007/s10689-021-00258-w

**Published:** 2021-04-28

**Authors:** Agata Pastorczak, Karolina Krajewska, Zuzanna Urbanska, Bartosz Szmyd, Elzbieta Salacinska-Los, Józef Kobos, Wojciech Mlynarski, Joanna Trelinska

**Affiliations:** 1 Department of Pediatrics, Oncology and Hematology, Medical University of Lodz, ul. Sporna 36/50, 91-738 Lodz, Poland; 2 Department of Surgery and Pediatric Oncology, Medical University of Lodz, Lodz, Poland; 3 Department of Histology and Embryology, Medical University of Lodz, Lodz, Poland

**Keywords:** Ovarian carcinoma, Children, Genetic predisposition, *SMARCA4*

## Abstract

Ovarian carcinoma is an extremely rare malignancy in children, often developing on the underlying inherited background. Female carriers of pathogenic germline mutations of *SMARCA4* are at risk of an aggressive type of undifferentiated ovarian cancer called small cell carcinoma of the ovary, hypercalcemic type (SCCOHT). Regardless of age of the patient, stage of the disease, and oncological treatment, the prognosis for SCCOHT is poor. Therefore, early intervention with risk-reducing surgeries is recommended for these patients*.* In this study, we report genetic testing of a family with two children carrying pathogenic germline mutations of *SMARCA4* and summarize the course of SCCOHT in all pediatric patients reported in the literature with constitutional defects identified within the *SMARCA4* locus.

**Supplementary Information:**

The online version contains supplementary material available at 10.1007/s10689-021-00258-w.

## Introduction

Pediatric ovarian neoplasms are rare tumors with a reported incidence of 2.6 per 100,000 girls [1]. Unlike in adult women, in whom epithelial carcinomas account for 90% of ovarian malignancies, germ cell tumors (GCTs) predominate in children [2]. Due to favorable histology of childhood ovarian tumors, pediatric patients exhibit an excellent 10-year survival rate of 85% [2]. However, some children (less than 5% of cases) develop tumors of epithelial origin, including also an extremely aggressive type of undifferentiated ovarian cancer called small cell carcinoma of the ovary, hypercalcemic type (SCCOHT) [1]. This neoplasm displays histopathological similarities to malignant rhabdoid tumors and is driven by somatic (57%) and/or germline (43%) inactivating mutations of the *SMARCA4* gene [3]. The SMARCA4 protein, which has helicase and ATPase functions, is a component of the ATP-dependent chromatin remodeling complex SNF/SWI, which is required for transcriptional activation of genes [4]. A particularly high rate of constitutional heterozygous mutations of *SMARCA4* is seen in pediatric patients who develop SCCOHT [3, 5–10]. These children are diagnosed with autosomal dominant rhabdoid tumor predisposition syndrome type 2 (RTPS2; OMIM #613325)[11], in which other malignant lesions are formed in the brain, spine, lung, bladder, pelvis, and kidney [12, 13]. Considering the rarity and poor prognosis of SCCOHT in children and the necessity for genetic counseling of affected families, we undertook this study on an affected family. In this study, we describe a family with two children that were carrying germline pathogenic mutations of *SMARCA4,* which encodes the BRG1 protein*.* One of them developed fatal ovarian carcinoma. Additionally, we summarize the course of SCCOHT in all pediatric patients with constitutional defects identified in the *SMARCA4* gene, as reported in the literature.

## Methods

Written informed consent for research was obtained from the patient’s parents. The study was approved by the Medical University of Lodz Review Board (No. RNN/108/18/KE) and was performed in accordance with the Helsinki Declaration. Clinical germline multi-gene panel testing was performed on peripheral blood DNA from the proband, and the detected variant was validated in peripheral blood DNA samples from the parents and the sister by in-house Sanger sequencing (Supplementary Methods). Surgical specimens of ovarian tumors from the patient were histologically examined and stained for SMARCA4 expression using rabbit monoclonal [EPNCIR111A] anti-BRG1 antibody (Abcam, UK). Loss of heterozygosity (LOH) analysis for the *SMARCA4* gene was performed on fresh ovarian tumor DNA using high-density SNP arrays (Supplementary Methods). We also analyzed all reports in the literature that describe ovarian cancer in patients below the age of 18 years, in whom pathogenic mutations within the *SMARCA4* gene were identified.

## Results

### Family report

**Fig. 1:**
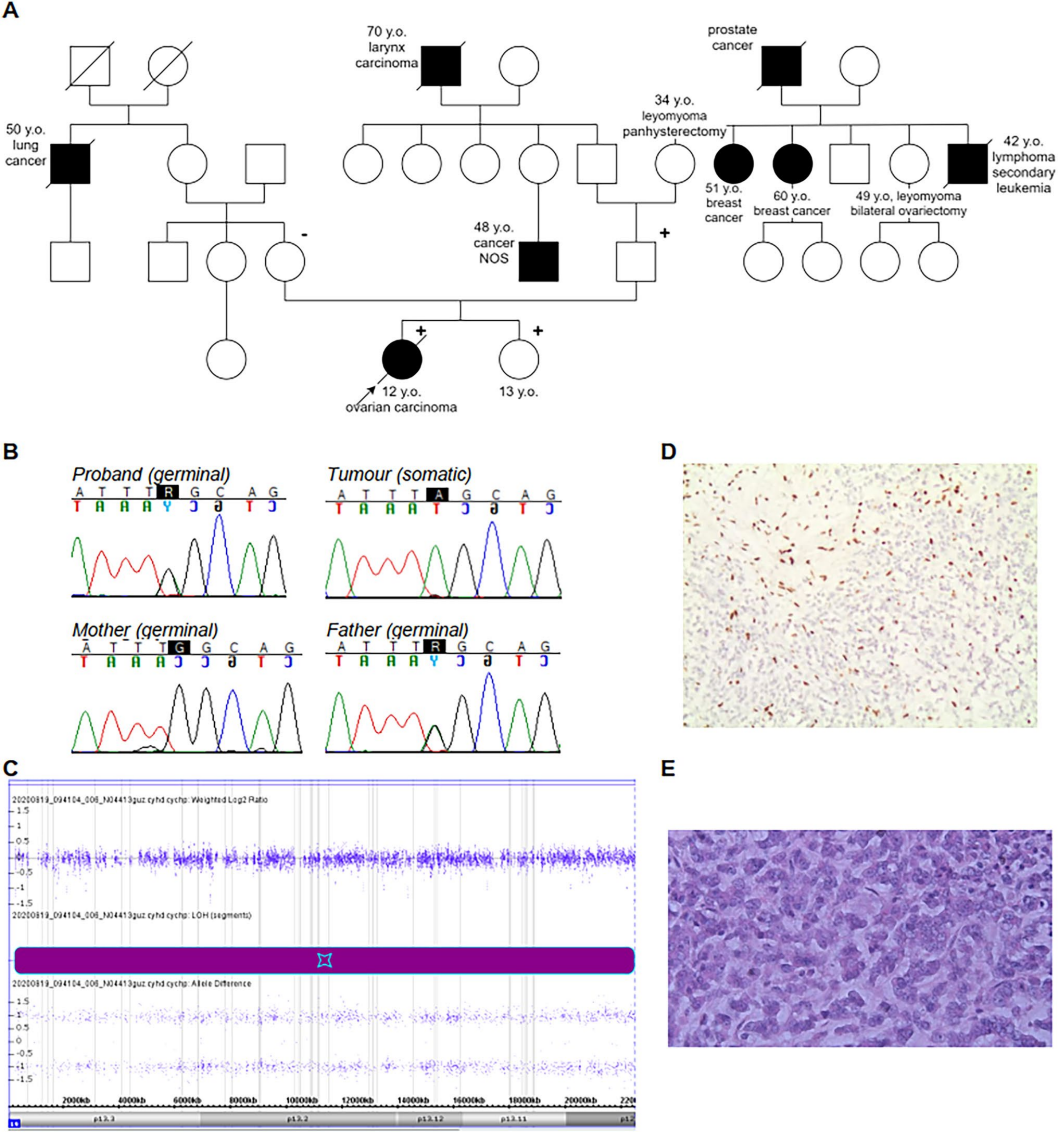
Genetic testing of the proband’s family. **a** A pedigree of the proband’s family. The proband with SCCOHT had a germline mutation in exon 24 of the *SMARCA4* gene (c.3310C > T) inherited from her father. This mutation was also identified in the proband’s sister. (+) denotes heterozygous mutation carrier in the germline; (−) denotes wild type in the germline. NOS denotes not otherwise specified. A diagonal line through a symbol indicates that the person is deceased. **b** Chromatograms of germline and somatic *SMARCA4* mutations in the proband and proband’s parents. **c** Results of single-nucleotide polymorphism array analysis of the tumor genome in the patient with germline *SMARCA4* mutation; Chromosome plot displays the presence of a loss of heterozygosity (LOH) at the 19p12-p13.3 region encompassing the *SMARCA4*. **d** Immunohistochemical staining of ovarian carcinoma using antibodies against BRG1 (100×). Loss of BRG1 expression was observed in 80% of cancer cells in the tumor. **e**.Hematoxylin–eosin staining of ovarian carcinoma of the proband (400×)

The proband, a fourteen-year-old girl, was admitted to the hospital due to weakness, weight loss with accompanying abdominal distention, and a palpable tumor in the hypogastrium. Serum alpha fetoprotein (AFP) and beta human chorionic gonadotropin (β-hCG) levels of the patient were normal, but cancer antigen 125 (CA-125) level was increased 40-fold. Magnetic resonance imaging revealed the presence of a tumor in the pelvis, which arose from the right ovary and exhibited dimensions of 16 × 14 × 11 cm along with ascites. There was no evidence of distant metastases. The histopathological results of the surgical biopsy indicated the presence of a large cell endocrine carcinoma that was part of the teratoma. Histopathological examination indicated the presence of small nests, cords, and trabeculae of neoplastic cells with scant eosinophilic cytoplasm and hyperchromatic nuclei **(**Fig. 1e). The patient received three courses of chemotherapy according to the TGM 95 protocol (VIP cycle: etoposide, ifosfamide, cisplatin) followed by macroscopic radical surgical removal of the residual mass and two postoperative VIP courses. Disease recurrence was noted one month later, when the patient underwent emergency operation because of suspected mesenteric torsion. At that time, two tumors in the pelvis and abdomen as well as massive neoplastic infiltration of the peritoneum were observed. After using extended panel of immunohistochemical staining tumor cells showed the following pattern of expression: vimentin-(+), synaptophysin—(+), MCA (+)-, EMA(+), INI-1 (+), CD56 (+), β-catenin (+), NSE (+), SMA (+), CD30 (−), LCA (−), AFP (−), CD117 (−), CD10 (−), S-100 (−), CD99 (−), CD68 (−), and desmin (−). The histopathological image and immunohistochemical stains were the most consistent with neuroendocrine carcinoma or a poorly differentiated neuroendocrine tumor. After receiving 1 cycle of chemotherapy (bleomycin, carboplatin, doxorubicin) followed by peritoneal drainage, no significant reduction in tumor size was seen. Due to massive peritoneal carcinomatosis, palliative care was administered. The patient died 8 months after the initial diagnosis of ovarian tumor.

The family history of the patient was examined, and a paternal grandmother and her sister who underwent panhysterectomy and salpingo-oophorectomy at the age of 34 and 49 years, respectively, were identified. Two other paternal aunts were diagnosed with breast cancer at the age of 51 and 60 years, respectively. A paternal uncle was diagnosed at the age of 34 years with lymphoma and secondary leukemia, and a paternal great grandfather developed prostate cancer (Fig. 1a).

The proband’s diagnosis was based on histopathological analysis, whereas other oncological diagnoses in her family were based on self-reporting by the proband’s mother.

Since such a rare, atypical histopathology of the tumor, accompanied by an aggressive course of the disease in the proband, was observed and the family history suggested more than one cancer predisposition gene, cancer gene panel testing was recommended for the patient (Supplementary Table S1). A pathogenic nonsense variant within exon 24 of *SMARCA4* gene [(NM_001128849.3):c.3310C > T,p.Gln1104Ter] was identified in the heterozygous form in the proband. This mutation was also seen in the father (Fig. 1b) and the thirteen-year-old asymptomatic sister. Microarray analysis of the somatic tumor tissue revealed a 21.185 kbp loss of heterozygosity (LOH) within the 19p12-p13.3 region (260912_22445808), which encompasses 692 genes including *SMARCA4* (Fig. 1c). Immunohistochemistry demonstrated complete loss of BRG1 expression in 80% of cancer cells within the tumor (Fig. 1d).

According to the will of the parents increased surveillance for the detection of early cancer using both biomarkers and sonography, every three months was applied in unaffected sister. Parents were informed about the limitations of such approach and the benefits of possible prophylactic bilateral oophorectomy preceded by the oocyte cryopreservation when the girl reaches puberty. Psychological support was offered to the family during the illness of the first child and after the genetic background of cancer was identified in the family members.

### Clinical phenotype of pediatric patients with germline mutation of *SMARCA4*

We analyzed the clinical phenotypes and genotypes of all the pediatric patients with confirmed germline mutation of *SMARCA4* reported in the literature and identified thirteen such cases (Table 1) [3, 6, 7, 9, 10, 14–16]. Nine of them carried constitutional nonsense mutations of *SMARCA4,* and LOH at 19p13.2 within the *SMARCA4* locus was found in somatic tissue in the majority of malignancies. Patients developed SCCOHT at a median age of 12 (10.5–14.5) years. Half of them showed hypercalcemia and increased serum levels of CA-125. Four patients had a family history of ovarian tumors in women below the age of 35 years. The therapeutic approaches used differed between patients and included chemotherapy, radiotherapy, and stem cell transplantation (in one case). Six out of thirteen patients died due to disease progression and one patient experienced fatal toxic complications after autologous bone marrow transplant [16].

**Table 1:** Clinical and biological characteristics of the pediatric patients carrying germline pathogenic mutation in the *SMARCA4* gene

n	Age at cancer diagnosis	SMARCA4 (NM_001128849) germline mutation	Mutation type	SMARCA4 somatic mutation/LOH	Serum CA-125	Stage at diagnosis	Hypercalcemia at diagnosis	Family history of cancer	Treatment	Outcome	Source
1	13	c.2935C > T, p.Arg979*	Nonsense	c.1236delC, p.Gln413Argfs*88	ND	IIIc	Present	No	ND	in CR at 2 years from diagnosis	[14]
2	12	c.898C > T, p.Gln300Ter	Nonsense	c.3215 + 1G > A	ND	ND	ND	ND	ND	ND	[6]
3	15	c.300delAG, p.Gly102Ter	Nonsense	LOH	Increased	IV	PRESENT	ND	PAVEP-CARBOPEC regimen	Deceased	[7]
4	12	c.2617-3C > G	Splice-site	LOH	Increased	ND	PRESENT	fatal SCCOHT in 24 ys. old mother and her 26 ys. old sister	PEB + RT	in CR at 3 years from diagnosis	[10]
5	11	c.4071 + 1G > A	Splice-site	LOH	Slightly increased	Ic	present	fatal SCCOHT in 23 years old mother	5 cycles PE/CE, at recurrence 1 cycles of VAE, 3 cycles of CX + RT	Deceased	[9]
6	15	c.643C > T, p.Gln215*	Nonsense	c.1687_1700del, p.Asn563GlyfsTer83	Normal	III	absent	Yolk sac tumor in mother at the age of 16 ys	4 cycles BEP, at recurrence 6 cycles XC + RT	Deceased	[3]
7	13	c.3239G > A, p.Gly1080Asp	Missense	c.1326delC, p. Ser442Argfs*59	Increased	I	present	No	6 cycles of XC	in CR at 1 years from diagnosis	[3]
8	7	c.1141C > T, p.Arg381*	nonsense	LOH	ND	IIc	ND	ND	ND	Deceased	[3]
9	18	c.2932C > T, p.Arg978*	Nonsense	LOH	ND	Ic	ND	ND	ND	Deceased	[3]
10	9	c.2935C > T, p.Arg979*	Nonsense	ND	ND	IA	present	ND	ND	ND	[15]
11	10	c.722_735delGTCCCGGCCCGGCA, p.Gly241fs	Frameshift	Homozygous c.722_735delGTCCCGGCCCGGCA, p.Gly241fs	ND	IIIC	present	ND	ND	ND	[15]
12	12	c.1141C > T, p.Arg381Ter	Nonsense	LOH	Increased	ND	absent	ND	EIP, followed by XCV, and + autoSCT	Deceased 5 months from diagnosis	[16]
13	14	c.3310C > T,p.Gln1104Ter	Nonsense	LOH	Increased	IIIa	ND	Grandmother and her sister underwent panhysterectomy and salpingo-oophorectomy at the age of 34 ys. and 49 ys	3 cycles of EPI, at recurrence 1 cycle of BCD	Deceased	Currently reported case

* (asterisk) indicates translation termination due to the presence of stop codon in coding sequence

*A* doxorubicin, *B* bleomycin, *c* carboplatinum, *D* adriamycin, *E* etoposide, *I* ifosfamide, *ND* no data, *P* cisplatinum, *RT* radiotherapy, *V* vincristine, *X* palitaxel, *V* bevacizumab, *CR* complete remission, *autoSCT* autologous stem cell transplantation, *ys* years

## Discussion

Pediatric ovarian neoplasms occur very rarely. However, in such cases, cancer predisposition syndromes should be considered, even if the tumor is not of epithelial origin. Several genetic disorders, including DICER1 syndrome, Peutz–Jeghers syndrome, Ollier disease, Maffucci syndrome, and WT-1 related syndromes, are associated with an increased risk for germ cell tumors in children [1]. However, the identification of such disorders does not necessarily affect the treatment and surveillance of patients and family members. In contrast, the diagnosis of RTPS2 in a child with ovarian cancer is essential for appropriate clinical management and genetic counseling [17].

RTPS2 is an autosomal dominant genetic disorder that predisposes infants and children below three years of age to extremely aggressive malignant tumors [17]. It is caused by a germline mutation of *SMARCA4*, which acts like a tumor suppressor gene. Most of the reported individuals diagnosed with *SMARCA4*-related RTPS inherited a pathogenic variant of this gene from an unaffected parent. The penetrance appears to be incomplete, and the types of RTPS-related tumors differ between members of the same family [13, 17]. Although *SMARCA4* mutations are less penetrant for atypical teratoid rhabdoid tumor than *SMARCB1,* SCCOHT was exclusively observed in patients constitutionally carrying the deleterious variant of *SMARCA4* [12]. In pediatric patients who developed SCCOHT, a family history of ovarian neoplasms at a young age and dismal outcomes were frequently observed.

SCCOHT is usually diagnosed in young women in the second or third decade of life [6]. In pediatric population, this tumor develops mainly in adolescents but individuals diagnosed below the age of 10 years have been also reported [6] [18]. Only 20% of pediatric ovarian cancers are of epithelial origin, and these exhibit a predominance of serous and mucinous histology [1]. SCCOHT is the rarest type in this group*,* and it is caused by somatic and/or germline *SMARCA4* mutations [19]. In this type, cancer cells resemble rhabdomyoblasts, which are small and round with hyperchromatic nuclei and are immunohistochemically characterized by the increased expression of vimentin, epithelial membrane antigen, and cytokeratins, as well as by the loss of BRG1 protein expression [20]. Additionally, 62% of adult patients and a similar percentage of children exhibit hypercalcemia when diagnosed with SCCOHT [6]. Usually, genetic testing is recommended when ovarian neoplasm with such characteristics is diagnosed in a child and it is eventually followed by the diagnosis of RTPS2.

SCCOHT is refractory to conventional oncological treatment in 75% of patients regardless of the patient’s age or stage of the disease, and median survival time does not exceed fifteen months. Despite individualized and diverse approaches for treatment, recurrence and progression of cancer have been observed in children diagnosed with SCCOHT. Novel therapies targeting the SCCOHT vulnerabilities [such as EZH2 and HDAC, bromodomain and extra-terminal motif containing protein inhibitors (BETi), tyrosine kinase, and PD-1 inhibitors] have been successfully tested, mainly in preclinical studies, but prospective multicenter protocols are highly needed in this particularly rare disease [18]. To this purpose, in 2018, the International SCCOHT Consortium was formed. It aims to explore the biology of SCCOHT in order to discover new targeted therapy and to identify an effective screening procedure among *SMARCA4* mutation carriers (https://www.smallcellovarian.org/consortium.html).

In the family examined in the present study, parents were offered genetic testing for their child because of the unusual histopathology of the tumor and a family history that indicated an inherited cancer predisposition. Although the profile of malignancies and age at onset among family members partly matched the phenotype of *BRCA1*- and *BRCA2*-associated hereditary breast and ovarian cancer, diagnosis of ovarian neoplasm in the proband at an early age, undifferentiated structure of the tumor, and expression of neuroendocrine markers were uncommon for this type of cancer.

Histopathological analysis revealed a large cell endocrine carcinoma that was a part of the teratoma, but the clinical course of the disease was atypical for a GCT. While both the serum markers, AFP and β-hCG, were not elevated, CA-125 levels were increased. Such a profile is indicative of epithelial ovarian cancers rather than GCT [21]. Additionally, cancer remission was short-lived, and recurrence was noted one month after the chemotherapy was completed. Although the patient showed a partial response after first-line platinum-based chemotherapy, which enabled the surgical removal of the residual mass, second-line chemotherapy was completely ineffective. Poor outcome in this patient is consistent with those of pediatric patients with germline mutations of *SMARCA4* that were previously described in the literature (Table 1). Recent studies have demonstrated that a combination of surgery, radiotherapy, and high-dose chemotherapy with stem cell rescue could be an effective approach for treating SCCOHT [22]. The administration of such aggressive treatments can only be justified by proper histopathological diagnosis[17]. However, pathologists often struggle with these tumors due to overlapping morphology and immunohistochemistry in this group of cancers [18].

Dismal outcome of pediatric SCOOHT raises questions regarding the optimal timing for genetic testing and early interventions with risk-reducing surgeries. Parents of an unaffected child from the described family decided for increased surveillance for detection of early cancer. However, neither biomarker nor sonography is an effective method for early diagnosis of ovarian cancer. Considering extremely aggressive course and high lethality of SCCOHT, *SMARCA4* mutation carriers must be counseled about prophylactic bilateral oophorectomy. There are no official recommendations regarding the age of genetic testing and prophylactic salpingo-oophorectomies for females carrying the pathogenic variant of *SMARCA4.* Prophylactic bilateral salpingo-oophorectomy in 13-year-old healthy carrier of germline *SMARCA4* mutation has been reported. In this case, histopathological examination of the ovaries and tubes, which were serially sectioned, revealed lack of malignant transformation [23]. Podwika et al*.* argued that, similar to the strategy applied to patients with Li-Fraumeni syndrome (germline *TP53* mutation) and Swyer syndrome (46, XY, gonadal dysgenesis), genetic testing should be initiated below the age of 18 years, preferably between the ages of 9 and 15 years [19]. This approach will enable early surgical intervention and allow preservation of fertility in the form of assisted reproductive technology. For female carriers of deleterious *SMARCA4*, all experts recommend bilateral salpingo-oophorectomy outside the pediatric age range and after completion of puberty, along with regular screening using abdominal ultrasound every six months during childhood [17, 19]. However, due to exceedingly poor outcome of the disease and lack of an effective screening strategy, prophylactic surgery in children below 18 years may be considered if the patient and the family express a strong desire to prevent cancer by surgical removal of the ovaries and tubes. Since the median age at cancer diagnosis in *SMARCA4* mutation carriers is lower as compared to *BRCA1/BRCA2* mutation carriers, the prophylactic surgery should be offered at least earlier, preferably below the age of 22. At that age, most of the patients do not consider conception yet. Therefore, the oocyte cryopreservation for in vitro fertilization accompanied by pre-implantation genetic testing which will prevent the transmission of pathogenic variant should be recommended as a complementary procedure to invasive surgery. However, genetic testing and prophylactic surgery in at-risk families should be preceded by consent and assent procedures that enable family members to estimate the risks and benefits of these tests and surgeries.

## Conclusions

SCOOHT is a rare tumor in pediatric population characterized by extremely poor outcome and frequent inherited background. Awareness of the disease should increase since propter pathological diagnosis and age-assisted decision-making regarding an optimal treatment approach are challenging.

## Supplementary Information

Below is the link to the electronic supplementary material.

Supplementary file1 (DOCX 59 kb)

## Data Availability

If requested.

## References

[1] Lala SV, Strubel N (2019). Ovarian neoplasms of childhood. Pediatr Radiol.

[2] Lockley M, Stoneham SJ, Olson TA (2019). Ovarian cancer in adolescents and young adults. Pediatr Blood Cancer.

[3] Witkowski L, Carrot-Zhang J, Albrecht S (2014). Germline and somatic SMARCA4 mutations characterize small cell carcinoma of the ovary, hypercalcemic type. Nat Genet.

[4] Stanton BZ, Hodges C, Calarco JP (2017). Smarca4 ATPase mutations disrupt direct eviction of PRC1 from chromatin. Nat Genet.

[5] Bailey S, Murray MJ, Witkowski L (2015). Biallelic somatic SMARCA4 mutations in small cell carcinoma of the ovary, hypercalcemic type (SCCOHT). Pediatr Blood Cancer.

[6] Connor YD, Miao D, Lin DI (2020). Germline mutations of <em>SMARCA4</em> in small cell carcinoma of the ovary, hypercalcemic type and in SMARCA4-deficient undifferentiated uterine sarcoma: clinical features of a single family and comparison of large cohorts. Gynecol Oncol.

[7] Lavrut P-M, Le Loarer F, Normand C (2016). Small Cell carcinoma of the ovary, hypercalcemic type: report of a bilateral case in a teenager associated with SMARCA4 germline mutation. Pediatr Dev Pathol.

[8] Moes-Sosnowska J, Szafron L, Nowakowska D (2015). Germline SMARCA4 mutations in patients with ovarian small cell carcinoma of hypercalcemic type. Orphanet J Rare Dis.

[9] McDonald JM, Karabakhtsian RG, Pierce HH (2012). Small cell carcinoma of the ovary of hypercalcemic type: a case report. J Pediatr Surg.

[10] Martinez-Borges AR, Petty JK, Hurt G, Stribling JT, Press JZ, Castellino SM (2009). Familial small cell carcinoma of the ovary. Pediatr Blood Cancer.

[11] Hasselblatt M, Gesk S, Oyen F (2011). Nonsense Mutation and Inactivation of SMARCA4 (BRG1) in an Atypical Teratoid/Rhabdoid Tumor Showing Retained SMARCB1 (INI1) Expression. Am J Surg Pathol.

[12] Agaimy A, Foulkes WD (2018). Hereditary SWI/SNF complex deficiency syndromes. Semin Diagn Pathol.

[13] Schneppenheim R, Frühwald MC, Gesk S (2010). Germline nonsense mutation and somatic inactivation of SMARCA4/BRG1 in a family with rhabdoid tumor predisposition syndrome. Am J Hum Genet.

[14] Errichiello E, Mustafa N, Vetro A (2017). SMARCA4 inactivating mutations cause concomitant Coffin-Siris syndrome, microphthalmia and small-cell carcinoma of the ovary hypercalcaemic type. J Pathol.

[15] Ramos P, Karnezis AN, Craig DW (2014). Small cell carcinoma of the ovary, hypercalcemic type, displays frequent inactivating germline and somatic mutations in SMARCA4. Nat Genet.

[16] David MP, Venkatramani R, Lopez-Terrada DH, Roy A, Patil N, Fisher KE (2018). Multimodal molecular analysis of an atypical small cell carcinoma of the ovary, hypercalcemic type. J Mol Case Stud..

[17] Foulkes WD, Kamihara J, Evans DGR (2017). Cancer surveillance in Gorlin syndrome and rhabdoid tumor predisposition syndrome. Clin Cancer Res.

[18] Tischkowitz M, Huang S, Banerjee S (2020). Small-cell carcinoma of the ovary, hypercalcemic type-genetics, new treatment targets, and current management guidelines. Clin Cancer Res.

[19] Podwika SE, Jenkins TM, Khokhar JK, Erickson SH, Modesitt SC (2020). Optimal age for genetic cancer predisposition testing in hereditary SMARCA4 Ovarian Cancer Families: How young is too young?. Gynecol Oncol Rep.

[20] Saunders J, Ingley K, Wang XQ (2020). Loss of BRG1 (SMARCA4) immunoexpression in a pediatric non-central nervous system tumor cohort. Pediatr Dev Pathol.

[21] Heo SH, Kim JW, Shin SS (2014). Review of ovarian tumors in children and adolescents: radiologic-pathologic correlation. Radiographics.

[22] Pressey JG, Kelly DR, Hawthorne HT (2013). Successful treatment of preadolescents with small cell carcinoma of the ovary hypercalcemic type. J Pediat Hematol.

[23] Pejovic T, McCluggage WG, Krieg AJ (2019). The dilemma of early preventive oophorectomy in familial small cell carcinoma of the ovary of hypercalcemic type. Gynecol Oncol Rep.

