# Supplementary material for: Ovarian carcinoma in children with constitutional mutation of SMARCA4: single-family report and literature review
Source: Fam Cancer. 2021 Apr 28;20(4):355–62. doi: 10.1007/s10689-021-00258-w (PMC8484133; doi:10.1007/s10689-021-00258-w)

**SUPPLEMENTARY MATERIALS**

**DNA isolation**

Genomic DNA was extracted from bone marrow and peripheral blood samples using the QIAamp DNA Blood mini kit (QIAGEN, Hilden, Germany). The concentration and quality of isolates were determined by ultraviolet spectrophotometry (NanoDrop 8000, Thermo Scientific, Waltham, MA).

**Microarray analysis**

Copy-number variation and LOH analysis of tumor tissue was performed using CytoScan HD array (2 670 000 probes including 750 000 SNP’s; Applied Biosystems, Thermo Fisher, Waltham, MA). In total, 250 ng of the genomic DNA was processed according to the manufacturers' protocol following the steps of digestion by NspI, amplification using a ligation-mediated PCR with adapters linked to the restriction fragments, purification of PCR products using magnetic beads, fragmentation using DNase I, labeling with biotin and hybridization overnight (16–18 h) to a 49-format array. After incubation samples were washed and stained with streptavidin using a GeneChip Fluidics Station 450 and were scanned by GeneChip Scanner 3000, using the GeneChip Command Console Software (Thermo Fisher Scientific), to generate the CEL files that includes the intensity probe signals. CEL files were then analyzed using the Chromosome Analysis Suite (ChAS) software (Thermo Fisher Scientific, Inc) and converted to CYCHP files containing information on copy number, loss of heterozygosity (LOH), mosaicism, and genotype calls. Copy number state and SNP genotypes were called using the Hidden Markov Model (HMM) algorithm and the Bayesian Robust Linear Model with the Mahalanobis distance classifier (BRLMM) algorithm, respectively. The intensity ratio of each SNP and CN probe in the DNA test provide a relative copy number (log2 ratio: log2sample − log2reference) which is normalized respect to a reference. The Reference Model File contains 380 samples, 284 from HapMap and 96 from BioServe Biotechnologies (BioServe Biotechnologies, Ldt; Beltsville, MD, USA). Determination of log2 ratio indicated if there is a gain or loss of genetic material. The minimal number of probes was applied to determine the CNAs: 50 probes for duplication (gain); 25 probes for deletion (loss). To further identify the genes involved in the CNVs, two databases were applied: the UCSC database (http://genome.ucsc.edu) and Ensemble (<http://www.ensembl.org>).

**Panel-based DNA sequencing**

Targeted NGS sequencing of leukemic genome was performed using custom designed SureSelect XT-HS2 DNA System panel (Agilent Technologies, USA) comprising 660 genes related to hematological diseases including *SMARCA4* gene. 10 ng of DNA was processed according to the manufacturers' protocol. The DNA libraries were prepared according to manufacturer’s protocols and sequenced on a Next Seq 550 system (Illumina, USA) in the process of 300 bp paired-end run. The raw data analyses were performed using SureCall v. 4.1 software and mapped to the GRCh37/hg19 reference sequence. The presence of the pathogenic nonsense variant within exon 24 of *SMARCA4* gene [[(NM_001128849.3):c.3310C>T](https://varsome.com/variant/hg19/SMARCA4(NM_001128849.3):c.3310C%3ET),p.Gln1104Ter] was confirmed by Sanger sequencing using the following primers: SMARCA4 :F- 5’CTGGACCTGTACCGAGCCTC - 3’ ; SMARCA4 :R - 5’ GTGCCGAGTGCCCAAGATG - 3’.

**Supplementary Figure 1**

The structure of the SMARCA4 protein (UniProt, SMCA4_HUMAN) overlaid with the alterations identified in 13 SCCOHT pediatric cases  (case numbers in parentheses). SNF2_N, SNF2 family N-terminal domain; helicase_C, helicase-conserved C-terminal domain; SnAC, Snf2-ATP coupling, chromatin- remodeling complex; bromo, bromodomain. Genomic coordinates for splice-site mutations can be found in Table 1. Numbers across the bottom of the schematic represent amino acids.


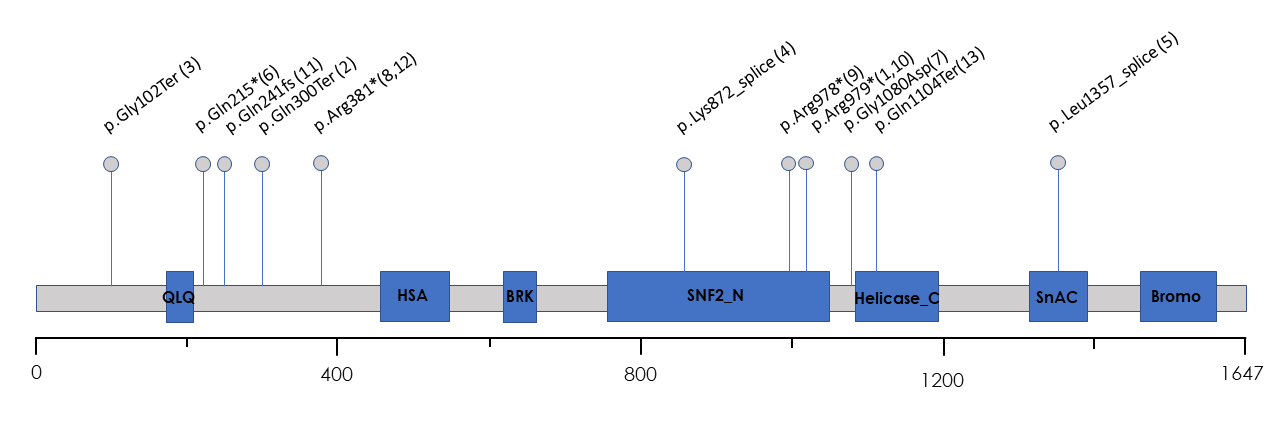

Supplement: Supplementary file 1 — Supplementary file1 (DOCX 59 kb) [file 10689_2021_258_MOESM1_ESM.docx]
